# From Structure to Phenotype: Impact of Collagen Alterations on Human Health

**DOI:** 10.3390/ijms19051407

**Published:** 2018-05-08

**Authors:** Lavinia Arseni, Anita Lombardi, Donata Orioli

**Affiliations:** 1 Department of Molecular Genetics, German Cancer Research Center (DKFZ), 69120 Heidelberg, Germany; l.arseni@dkfz-heidelberg.de; 2 Istituto di Genetica Molecolare, Consiglio Nazionale delle Ricerche, 27100 Pavia, Italy; anita.lombardi@igm.cnr.it

**Keywords:** collagen, extracellular matrix, skin defects, bone fragility, muscle weakness

## Abstract

The extracellular matrix (ECM) is a highly dynamic and heterogeneous structure that plays multiple roles in living organisms. Its integrity and homeostasis are crucial for normal tissue development and organ physiology. Loss or alteration of ECM components turns towards a disease outcome. In this review, we provide a general overview of ECM components with a special focus on collagens, the most abundant and diverse ECM molecules. We discuss the different functions of the ECM including its impact on cell proliferation, migration and differentiation by highlighting the relevance of the bidirectional cross-talk between the matrix and surrounding cells. By systematically reviewing all the hereditary disorders associated to altered collagen structure or resulting in excessive collagen degradation, we point to the functional relevance of the collagen and therefore of the ECM elements for human health. Moreover, the large overlapping spectrum of clinical features of the collagen-related disorders makes in some cases the patient clinical diagnosis very difficult. A better understanding of ECM complexity and molecular mechanisms regulating the expression and functions of the various ECM elements will be fundamental to fully recognize the different clinical entities.

## 1. Introduction

The extracellular matrix (ECM) is a non-cellular complex network that provides a structural scaffold to the surrounding cells and, at the same time, a deposit of cytokines and growth factors capable of influencing cell behaviour.

The main ECM components include collagens, proteoglycans and glycoproteins [1]. In addition, many proteins such as growth factors, cytokines and proteolytic enzymes are associated to the ECM. Through all these components the ECM provides environmental information to the cells, which in turn respond by adapting their behaviour and adjusting proliferation, migration and differentiation. The ECM is highly dynamic and its remodelling has to be tightly regulated in order to maintain tissue homeostasis. Deregulation of the ECM structure or composition contributes to the onset of a variety of pathological conditions characterized by a wide range of tissue alterations, further straightening the functional relevance of the ECM. After a general overview of the ECM main features and functions we will focus on the most abundant elements of the ECM, the collagens. We will discuss the different collagen types, their synthesis and structural organization as well as the relevance of properly assembled collagen fibres by discussing their impact on human health.

## 2. The ECM: Molecular and Structural Diversity

The ECM is a highly dynamic and heterogeneous structure. Each tissue has an ECM with a unique composition generated in early embryonic stages and then maintained and remodelled throughout the entire life. The great complexity of ECM structure and functions makes the research in this field very challenging. A significant input derives from the definition of the “matrisome,” a list of proteins contributing to the ECM in different organisms and tissues, which has been predicted by integrating the information derived from experimental knowledge, genome comparative studies and bioinformatics tools [2]. This plastic and evolving list of ECM proteins, comprising between 1% and 1.5% of the mammalian proteome, requires further investigations and biochemical characterization [3].

### 2.1. Chemical Composition and Mechanical Properties

**Figure 1:**
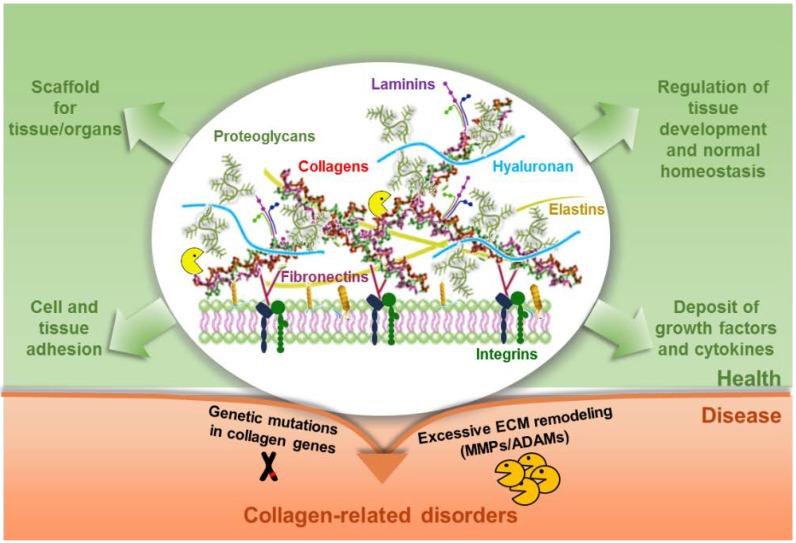
Schematic representation of the complex meshwork of proteins forming the extracellular matrix (ECM). The main ECM components, namely collagens, proteoglycans, hyaluronan, fibronectin, laminin and elastin, as well as the integrin ECM receptors, are depicted. The ECM provides mechanical support and anchoring for cells and tissues but it also acts as a reservoir of growth factors and cytokines and regulator of normal tissue development and homeostasis. Alterations in any of these functions result in a pathological status characterized by various tissue abnormalities.

Hundreds of ECM components are known, many of which are capable of binding to other ECM components on specific sites, thus making the matrix a highly intricate structure (Figure 1). 

Although the molecular composition can vary widely, the ECM main components are: proteoglycans, hyaluronan, adhesive glycoproteins (fibronectins and laminins) and fibrous proteins (collagens and elastin). Proteoglycans are constituted by large carbohydrates (generically referred as glycosaminoglycans, GAGs) attached to a protein core. The anionic polysaccharides GAGs allow the sequestration of water and other cations such as calcium [3]. Several types of protein core exist and various types of GAGs can bind to each other in a wide variety of combinations. Proteoglycans promote cell-ECM adhesion but also bind to secreted proteins and growth factors in the ECM. Many components of this category have space-filling and lubrication functions. A special type of GAG is hyaluronan (HA), the only GAG element of the ECM that lacks a protein core. It is a linear polysaccharide composed of the repeating disaccharide units *N*-acetyl-d-glucosamine and d-glucuronate [4]. It is particularly abundant in human tissues, including joints, eyes, umbilical cord, synovial fluids, skeletal tissues, hurt and lung. In spite of its simple structure, HA plays a role in several biological processes, including cell signalling, inflammation, wound healing and cell development [5] and contributes to maintain tissue homeostasis by interacting with other ECM proteins or proteoglycans [6]. Thanks to its hygroscopic features, it acts as space filler among cells and contributes to the maintenance of tissue hydration. Its biocompatibility makes HA suitable for tissue engineering and clinical applications, where it can be used as a diagnostic marker [7].

In mammals, around 200 glycoproteins provide interactions with other ECM components thus allowing ECM formation and assembly. They share multiple repeating domains and motifs typical of the ECM constituents and promote cell adhesion, cell signalling and binding to growth factors [8]. The best-studied ECM glycoproteins are fibronectins and laminins. Fibronectins are proteins encoded by a single gene through multiple alternative splicing. The dimerization of two fibronectin monomers results in the final molecule, which contains repeating units named type I, II and III. Type I and II are tight together by disulphide bonds, the third one has seven-stranded β-barrel composition. All these modules are organized in order to contain binding sites for a variety of other molecules, such as heparin sulphate proteoglycans (HSPGs), integrins and collagens [9]. Fibronectins can be soluble or associated to fibrils thus acting as bridging factors among different ECM components and anchoring cells to matrix fibres. Laminins are large cross-shaped ECM proteins composed of α, β and γ chains. In particular, twelve genes encode 5α, 4β and 3γ chains, which can differentially combine to generate many types of laminins. Among these, laminin G domain-like (LG) of the laminin α2 chain is composed by a β sandwich structure and contains a calcium-binding site, surrounded by a large number of epitopes involved in the interaction with cellular receptors and extracellular ligands ([10,11] and references therein).

Fibrous proteins include collagens and elastin. Collagens are the most abundant proteins of the ECM and a detailed description of their structure and functions is found below (Section 4). Elastin is a key element of the ECM that provides elasticity and flexibility to different tissues including large arteries, ligaments, tendon, lung, skin and cartilage. It is synthesized and secreted as tropoelastin, a soluble precursor implicated in the formation of elastic fibres through the interaction with the N-terminal domains of fibrillins 1 and 2. Tropoelastin contains several hydrophobic domains (consisting in proline, glycine, valine and alanine) that are responsible for the extensibility properties of the protein. The tensile strength provided by collagen fibrils is therefore counterbalanced by the extensibility of elastic fibres, which, conversely to collagen, can undergo progressive stretching and relaxation cycles. Dynamic tissues are thus able to sustain mechanical stress without being permanently affected but reverting the tissues back to their original shape, a property known as viscoelasticity [12]. The ECM tensile strength is determined by the dynamic activities of lysil oxidase (LOX) and lysil hydroxylase, two enzymes involved in regulating the cross-linking between collagens and elastin [13].

### 2.2. ECM-Bound Growth and Secreted Factors

Although not structural components of the matrix, many growth factors can bind to elements of the ECM and therefore are categorized as ECM constituents. Indeed, it was shown that vascular endothelial growth factor (VEGF) binds to the type III modules of fibronectin and this interaction depends on the heparin-binding residues of fibronectin [14]. VEGF and fibroblast growth factor (FGF) can bind to HSPGs from where they are cleaved off as soluble ligands by the heparanase enzyme. Hepatocyte growth factor (HGF) binds to the 70 kDa N-terminal and the 40 kDa C-terminal fragments of fibronectin [15], whereas platelet-derived growth factor (PDGF) binds the type III and the variable domains of fibronectin [16]. Differently, transforming growth factor-beta (TGFβ) binds to the latent transforming growth factor beta binding protein (LTBPs), which in turn binds to fibrillins and fibronectin-rich matrices [14,15,17,18]. Also, fibrillin-containing microfibrils of the ECM regulate the availability and activity of bone morphogenetic proteins (BMPs) and growth and differentiation factor-5 (GDF-5), cytokines of the TGFβ family [19]. Overall, the different growth factors associated to the ECM could be released locally and become available for the interaction with their canonical receptors. Thus, the ECM serves as a storage for growth factors and chemokines, whose interactions with the matrix control their half-life, local concentration and biological activity.

## 3. ECM Functions

For many years, the ECM has been defined as a static structure whose unique function was to provide support and shape to cells and tissues. Although this passive role is definitively fundamental for organism organization and maintenance, it is now clear that the ECM is much more than simple scaffolding. Synthesized and organized by the cells, the matrix itself can actively regulate cell behaviour [20]. Indeed, the ECM provides a substrate over which cells can adhere and migrate by sensing the ECM constituents. In turn, cells will secrete new elements that result in ECM remodelling. Therefore, through the coordinated action of its main molecular constituents, the ECM can influence cell proliferation, adhesion and migration as well as differentiation and cell death [21].

### 3.1. Structural Roles of ECM 

The ECM plays important structural roles during development and in particular during the formation of the skeleton. In tissues with mechanical functions such as cartilage, bone and tendons, the ECM is the major component that confers structural properties.

Thanks to its biologically diverse array of macromolecules, the ECM provides a robust and dynamic scaffold capable to evolve during the normal physiological development but also to face insults that could disrupt tissue homeostasis. ECM reactions to these conditions are mediated by its physical, biochemical and biomechanical properties. Depending on its chemical composition, its topography and dimensionality, the ECM exerts different stimuli on the cells, which sense these forces and in turn respond to them. Cell migration, which is critical for proper normal embryonic development, is one of the best examples. Both motile (like the immune cells) and non-motile cells (such as adult epithelial cells) sense the composition and density of the surroundings and respond by migrating towards or moving away from the source.

Tissue elasticity also depends on the ECM chemical composition, which defines soft or stiff matrices. Human tumours for example are surrounded by a stiff matrix with high collagen concentrations and the ECM rigidity might represent an optimal growing milieu for some surrounding cells that are therefore attracted towards this source [22]. This phenomenon, known as desmoplasia, is usually associated with malignant tumours and it is present in many solid tumours. Tissue stiffness can drive malignant transformation via integrin-mediated mechanisms [23] and such fibrotic “stiff” lesions are associated with a poor prognosis [24].

ECM protein receptors, including integrin, syndecan, discoidin domain receptors (DDRs) and proteoglycans, can act synergistically to anchor the cells to the matrix and favour the reciprocal matrix organization. These adhesion dynamics are particularly important to maintain the right balance between self-renewal and differentiation of stem cells [25]. In this respect, it has been shown that the genomic loss of integrin β1 encoding gene in the basal cells of mouse mammary epithelium affects stem cell regeneration and results in irregular branching ducts due to developmental defects of the mammary gland [26].

### 3.2. Signalling Modulation

As previously mentioned, the ECM can sequester several growth factors that are not structural components of the matrix per se but become active elements of the ECM. Chemokines, cytokines and growth factors, such as VEGFs, Wnts and FGFs, can be retained by the ECM and therefore create a “reservoir” of signalling molecules. By retaining these factors, the ECM may preserve the ligand source in proximity of the receiving cells and prevent their diffusion to the extracellular space. In addition, through the adhesion with ECM components, the ECM can modulate the ligand-receptors interaction and control the formation of morphogen gradients, whose concentration regulates developmental processes [21]. One example of ECM molecule directly implicated in the regulation of morphogens gradients is represented by the HSPGs, which binds morphogens but also many cell surface co-receptors, thus acting as a linking platform that mediates the interactions of morphogens with the other ECM components [27].

The ECM also contributes to ligand maturation. One representative example is the TGFβ proteins, which indirectly connect to fibrillins and fibronectins [18] and are stored in the ECM in their inactive form until proteolitically activated by matrix metalloproteinases (MMP) or by mechanical forces. Furthermore, the ECM can trigger signalling events, as shown by the biologically active fragments derived from the proteolytic cleavage of collagens, proteoglycans, elastin and laminins ([28] and references therein). Matrikines were first described by Maquart and colleagues in 1999 [29] and then named matricryptins one year later by Davis and colleagues with the following definition “biologically active sites that are not exposed in the mature, secreted form of ECM molecules but which become exposed after structural or conformational alterations” [30]. Among them, endostatin derived from collagen XVIII [31,32] is the most extensively studied matricryptin. The ectodomains of membrane collagens XIII, XVII, XXIII and XXV [33] are matricryptins involved in cell adhesion, migration or proliferation [34]. Matrikines from collagens IV are involved in angiogenesis [35] and synapse formation [36]. The ectodomains of syndecans 1–4 are also matricryptins, whereas fragments derived from hyaluronan degradation regulate inflammation and wound healing ([28] and references therein). Therefore, these ECM-fragments may act by regulating cell proliferation, cell death, cell differentiation and angiogenesis [35]. Also, ECM receptors play a role in signal transduction, in particular the collagen receptors DDRs with their intracellular tyrosine kinase activity and the integrin proteins capable of transmitting chemical signals into the cells [37]. Upon ligand binding, receptors get activated and trigger intracellular signalling events that through the involvement of Rho, Rock and the pathway of MAP kinases modulate cellular survival, proliferation and differentiation [21]. 

### 3.3. ECM in Development

The ECM provides several different contributions to the developmental events where it plays a dual role, both as functional as well as structural supporting element. A well-known example is provided by the morphogens, soluble factors contributing to define the patterning of surrounding cells during embryonic development. Morphogens are produced in restricted areas of the embryo from which they diffuse and, thanks to the presence of the ECM, generate gradients of signalling molecules that influence cell migration, adhesion and contractility by the activation of intracellular signalling pathways. Meanwhile, the ECM behaves as a structural element by defining the roads for cell migration, delimiting differentiating tissues and maintaining the shape of developing organs. In this context, the ECM assumes architectural roles, such as insulation of tissue to avoid nonspecific adhesion between tissues or, conversely, mediating adhesion between different tissue layers. The opposite functions of insulating or gluing embryonic tissues together show the flexibility of the ECM, which can select one or the other or even synchronize both events according to environmental stimuli. An example of coexistence is observed during muscle differentiation, where a sticky ECM is required to bind the extremities of the cells, whereas a slippery matrix coats the lateral sides [38]. A slippery ECM is defined as an intact basement membrane (composed by laminin, integrin and glycoproteins) that allows tissues to freely slide on each other. GAGs with their negative charges and the resulting chain-chain repulsions, are the main cause of tissue slippery. Conversely, the removal of laminin induces a fragmentation of the basement membrane that, together with the presence of cell adhesion molecules (CAM), results in tissue sticking. 

ECM plasticity is also fundamental during branching morphogenesis of several vertebrate organs such as lung, kidney and mammary gland but also in skeletal development. During osteogenesis, skeletal progenitor cells undergo several morphological changes to ultimately give rise to the adult bone [39]. In mature bone, the ECM is the result of active and opposing remodelling events exerted by the osteoclasts and osteoblasts, which degrade and deposit the bone matrix, respectively. An imbalance between degradation and deposition leads to alteration of bone density and disease.

### 3.4. Cell Migration

The ability of cells to move is central for embryo development as well as maintenance of multicellular organisms. Cell movement strictly depends on the balance between adherence to and release from the ECM in a dynamic fashion. The adhesive properties of the cells are mainly regulated by integrins, which play both structural and signalling roles. Integrins can sense the physical state of the matrix, by interacting with specific ECM molecules, such as collagens and laminins and activate downstream intracellular signalling cascades involving the focal adhesion kinase (FAK) signalling pathway, the mitogen-activated protein (MAP) kinases and the Rho family GTPases [40,41]. Cells tend to migrate along oriented fibrils in a non-random movement. The removal of specific ECM components at a specific time, such as MMP-dependent proteolysis, can instead reorganize the ECM structure and therefore alter or promote the migration process. Thus, the ECM is not only a substrate but it plays dynamic and opposing roles in regulating cell migration. On one side, the basement membrane with its dense fibrillar protein network acts as a barrier to migrating cells, on the other, the ECM promotes cell movement by exposing chemotactic factors that can attract or repulse cells. The ECM remodelling contributes to the formation of organized pathways along which the cells can migrate in an oriented way. Collective cell migration along oriented patterns is an essential aspect of wound healing, a multi-steps process in which the skin repairs itself after injury. During this process, the ECM regulates the interactions between epidermal, dermal and bone marrow cells, it influences cell proliferation and orchestrates the deposition of new connective tissue and the migration of keratinocytes to the wound site.

### 3.5. ECM Remodelling 

To fulfil its activities, the ECM requires a constant and regulated remodelling whose precise orchestration is crucial for tissue homeostasis and developmental processes characterized by transient and dynamic signalling events. ECM remodelling implies changes in ECM composition (novel synthesis or degradation of specific ECM components) or ECM architecture (modification of the macromolecule organization). Several enzymes are involved in ECM remodelling, below is provided a short description of the most known and characterized enzymes.

Matrix metalloproteinases (MMPs) are a large family of enzymes that participate in the degradation of all major ECM components, including those of the basement membrane. They are zinc-dependent endopeptidases initially secreted in the extracellular environment as inactive zymogens with a pro-peptide domain that needs to be removed to allow enzyme activation. The MMPs can be either soluble or membrane-bound and present a substrate-specificity. At least 24 MMPs proteins have been so far identified and, based on their structural organization and substrate specificity, MMPs can be classified into: collagenases, gelatinases, stromelysins, matrilysins and membrane-type I [42]. Under physiological conditions MMP activities are tightly regulated but they may increase during pathological events.

Adamlysins, also called ADAMs (a disintegrin and metalloproteinases) and ADAMTS (ADAMs with a thrombospondin motif), are ECM proteinases involved in cell phenotype regulation, adhesion and migration. The ADAMs include both transmembrane and secreted proteins, whereas the ADAMTSs only contain secreted proteins. 21 ADAMs and 19 ADAMTS are known. They share several structural domains including the metalloproteinase as well as the disintegrin domain, the latest being involved in the binding to integrins. ADAMs are involved in cytokines processing and growth factor receptor shedding [43] while ADAMTS play a role in degradation of ECM components, particularly collagens and proteoglycans [44].

Meprins are membrane-bound or secreted metalloproteinases capable to cleave ECM molecules including the collagen type IV and fibronectins. In addition, they are involved in the synthesis of mature collagen molecules and in the activation of other metalloproteinases including MMPs and ADAMs [45].

The right balance between ECM degradation and deposition has to be guaranteed for the correct tissue integrity. It is therefore evident the relevance of ECM proteinase inhibitors. The tissue inhibitors of metalloproteinases (TIMPs) represent a small family composed of only four members with the function of reversibly inhibiting the activity of MMPs and ADAMs. TIMPs present two distinct domains, one at the N- and one at the C-terminal region of the protein, which are responsible for the binding and inhibition of MMP activity, respectively. Although each TIMP molecule is active against various MMPs, they all show some substrate preferences [46].

Other enzymes may be involved in ECM remodelling. Various proteolytic enzymes, including serine proteinases, cathepsins, heparanases, sulphatases and hyaluronidases, were shown to target ECM proteins. Plasmin and elastase are serine proteinases, the first degrades fibrin, fibronectin and laminin [47], whereas the latter degrades fibronectin and elastin [48]. Cathepsins are lysosomal proteases, which are appointed to the degradation of intracellular or endocytosed proteins. Under specific circumstances cathepsins can be secreted in the extracellular environment where they contribute to the ECM protein degradation. Heparanases and sulphatases cleave heparin sulphate [49] and remove its 6-*O*-sulphate residues, respectively. They affect the ability of heparin sulphate to bind several growth factors such as VEGF, PDGF and FGF, thus altering the downstream signalling events [50]. Hyaluronidases are a family of enzymes capable of degrading HA [51]. 

## 4. Collagens

Collagens are the major insoluble fibrous proteins in humans and other vertebrates, accounting for about a quarter of their total protein mass. So far 28 different types of collagens have been identified in vertebrates. They assemble to adopt a triple-helix conformation that gives rise to long thin fibrils or two-dimensional reticulum or even associate with other ECM elements. The different types of collagens and their structure are crucial to provide mechanical stability, elasticity and strength to tissues and organs.

### 4.1. Collagen Synthesis and Organization

**Figure 2:**
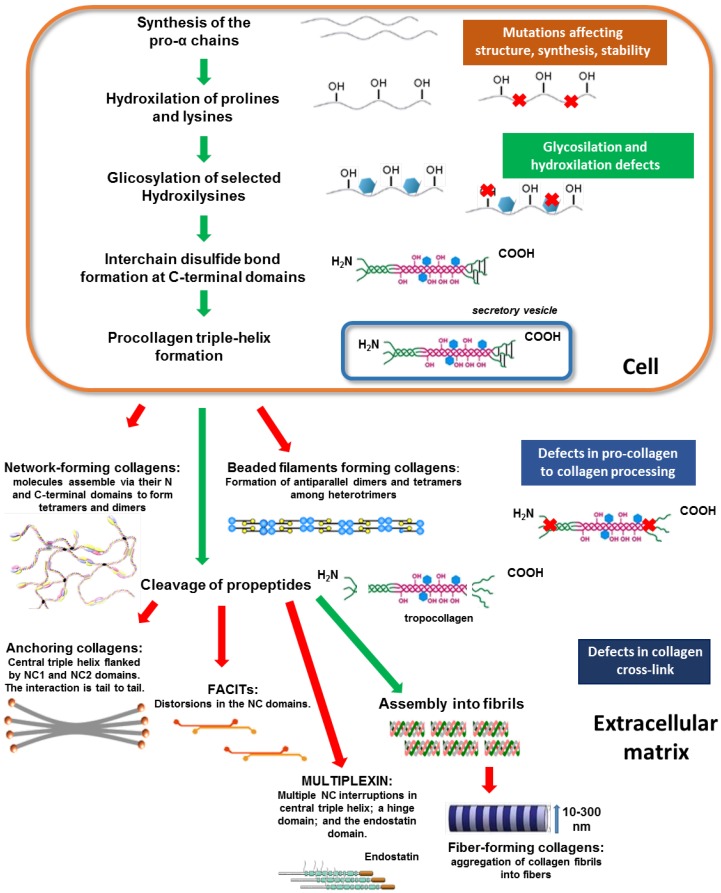
Schematic representation of collagen biosynthesis steps. The main biosynthesis steps of various collagen types are indicated. Green arrows highlight the contiguous processing steps whereas red arrows indicate the final step of collagen assembly into the different structural conformations. Coloured boxes on the right indicate potential alterations occurring during collagen processing at different steps, thus causing abnormalities in the structure and/or assembly. Abbreviations: NC, non-collagenous domain.

Fibrillar collagens are the most abundant collagens in humans and they are synthetized as long precursors, known as procollagens, which contain a large polypeptide extension at both the N- and C-terminal ends. The C-propeptide has an essential role inside the rough endoplasmic (ER) reticulum where it initiates the assembly of three coiled subunits (α chains) one around the other and along a central axis in order to generate right-handed triple-helix. In vertebrates, over 40 genes encode collagen α chains, which are differentially combined to form 28 different collagen types. Despite the different structural organization, all collagen types share the triple-helix structure. An essential element for the assembly of the three α chains is the proline-rich tripeptide Gly-X-Y repetition, which characterizes all collagens. In the triple helix, glycine residues are localised in the central part, thus allowing a close packing of the molecule [52,53]. Proline and hydroxyproline residues usually occupy the X and Y positions of the tripeptide. Moreover, hydroxylation of prolines and lysines in the middle region of the chains allows the formation of intra-molecular hydrogen bonds that stabilize the entire complex. The extent of lysine hydroxylation varies between tissues and collagen types. Some of the hydroxylysines are further modified by glycosylation with galactose and glucose [54]. Notably, the short N- and C-terminal portion of the chains, which do not assemble in the triple-helix, are required for the extracellular secretion of the polypeptide and the formation of collagen fibrils. The N- and C-propeptides (telopeptides) are subsequently removed by procollagen aminoproteinases and procollagen carboxyproteinases, respectively, giving rise to tropocollagen units [55,56]. Finally, adjacent tropocollagens are bound together through the formation of intermolecular interactions that involve lysine and hydroxylysine residues, thus providing the tensile strength of collagen fibrils. Finally, fibrils assemble into fibres of larger diameter [52]. Details on the synthesis of the other collagen types are included (Section 4.2) and shown in Figure 2.

### 4.2. Nomenclature and Classification

Collagens can be grouped based on their structure, function and tissue distribution. They are designated by Roman numerals according to the order of their discovery (I-XXVIII) [53]. They are formed by three identical chains (homotrimers) or by two/three different chains (heterotrimers). The most abundant collagen of the human body, the interstitial type I collagen, is made by two identical α1 and one α2 chain, which shows high sequence homology with α1 [57]. In most other cases, including the collagen type II, they are homotrimers made by three identical α1 chains. The length of the triple helical region differs among the various collagens. The tripeptide repetition is the predominant motif in fibril collagens whereas it is much shorter and frequently interrupted by non-triple helical domains in other collagen types (such as the non-fibrillar collagens). Non-collagenous (NC) regions may also have structural function as shown by the transmembrane collagens [58].

Following a classification based on collagen function and composition, we can distinguish:

Fiber-forming collagens: they are characterized by a fibrillar shape and a rope structure. Under electron microscopy they show characteristic banding pattern. Fibril collagens assemble to form fibres whose diameter ranges from 12 to >500 nm and the length varies depending on the tissue and developmental stage. They are stabilized by non-reducible covalent crosslinks among specific triple-helix domains and telopeptides [59]. They include the most abundant collagens of the organisms, such as the interstitial collagens (types I, II and III) and the collagen types V and XI, whose main functions consist in providing structural support, balance of pulling forces and enabling cell movement.

FACITs (fibril-associated collagens with interrupted triple helices): they contain short collagenous regions with interruptions in the triple helix intercalated by four NC regions. These molecules are mostly heterotrimers and carry a glycosaminoglycan side chain. They include collagen types IX, XII and XIV, which associate with various collagen fibrils.

Network-forming collagens: they are non-fibrillar collagens that aggregate linearly or laterally to form open networks. They are longer than classical collagens and can give rise to different kinds of networks depending on the collagen type. In particular, collagen type IV, the main component of epithelial basement membranes as well as vascular basal lamina, is irregularly assembled. Differently, collagen types VIII and X form regular hexagonal networks. The function of these network-forming collagens varies and likely depends on their structural organization [53,60].

Transmembrane collagens: they are expressed in many different tissues and cells. This group of collagens plays an important role in epithelial and neural cell adhesion as well as in epithelial–mesenchymal interaction during morphogenesis. They are characterized by the presence of several triple helical regions in the extracellular C-terminal domain interspersed by NC stretches. Next to the extracellular portion of the protein, there is a conserved coiled-coil domain essential for the trimerization of transmembrane collagens. Collagen types XIII and XVII are included in this group [61].

MULTIPLEXINs (multiple triple-helix domains and interruptions): collagen types XV and XVIII consist of several collagen domains with NC interruptions in the triple helixes, which are able to form oligomeric assemblies. They are found in some basement membranes covalently linked to glycosaminoglycan chains. The NC1 domain of collagen types XV and XVIII includes a peptide (endostatin) that following proteolytic cleavage is released in the extracellular environment. Several studies show the anti-angiogenic properties of endostatin as inhibitor of endothelial cell migration and tumour growth [62,63].

Anchoring fibrils: collagen type VII is the major component of the anchoring fibrils, whose function is to secure the adhesion of the epidermal and dermal layers. It consists of a central collagenous triple-helical domain flanked by NC1 and NC2 domains. The NC2 domain is proteolytically cleaved while NC1 is preserved to anchor other ECM elements, including collagens and laminins. The anchoring filaments are assembled in an antiparallel manner, tail to tail with some C-terminal overlap [62,64].

Beaded-filament-forming collagen: collagen type VI is the archetypal beaded filament-forming collagen. It is widely expressed and holds up tissue integrity. Collagen VI monomers are made up of short triple helical domains, which aggregate linearly to form beaded filaments or laterally through their globular domains, thus creating 3D networks. For this reason collagen type VI can also be included among the network-forming collagens [60]. The N and C non-collagenous regions of the monomers are preserved and antiparallel dimers and tetramers are assembled intracellularly [53,62].

Notably, collagen-like triple helical domains are found in several other proteins that do not have structural function and therefore are not considered as real collagens [65].

### 4.3. Collagen Degradation

Collagens have a great structural stability, resulting in high resistance against degradation by bacterial collagenases and other peptidases. Nevertheless, under physiological conditions most connective tissues undergo to a persistent turnover and continuous remodelling. Collagen degradation is a multi-step process that relies first on the activity of extracellular proteases to break down the ECM collagen fibrils and subsequently on the cellular uptake and intracellular lysosomal degradation of fragmented fibrils [66]. The extracellular fragmentation of collagens is mainly mediated by proteinases such as the MMPs (collagenases and stromelysin), cysteine cathepsins and serine proteinases (plasmin). MMPs can target a wide range of ECM proteins, not only collagens. They act at neutral pH and recognize specific cleavage sites on the target molecules [67]. Cathepsins are lysosomal proteases active at acidic pH, which can be active both intracellularly and upon secretion. Cathepsin S was shown to target and degrade collagens [68]. An indirect collagen digestion can be achieved in response to plasminogen activation to plasmin. The pro-MMP-2 enzyme is activated by plasmin into MMP-2, also known as gelatinase A. Upon activation, MMP-2 can degrade several collagen types, fibronectin, elastin as well as gelatin, the denatured form of collagen [69]. The extracellular collagen fragments are then recruited through phagocytosis from the neighbouring cells, mainly fibroblasts and macrophages, which send them to degradation via the lysosomal pathway [70]. The relationship between the extracellular and intracellular pathways is complex and not fully understood. It has been shown that in macrophages uPARAP/Endo180 acts as collagen internalization receptor after the interaction with pro-uPA (pro-urokinase plasminogen activator) and uPAR (urokinase plasminogen activator receptor [71]) proteins. Moreover, collagen internalization requires the expression of specific integrins and cytokines, including TGFβ and interleukin 1α. Finally, lysosomes fuse together to generate large structures containing collagen and ECM fragments that undergo enzymatic digestion by cysteine cathepsins [72,73].

## 5. Collagen Alterations in Pathological Events

The tight regulation of ECM synthesis and remodelling is fundamental for human health, as attested by the high number of hereditary disorders caused by mutations in genes encoding structural elements of the ECM or proteases implicated in the remodelling process. Alterations of ECM remodelling can also influence the course and progression of several other pathological conditions, including fibrosis, skin disorders and cancer [74]. The excessive ECM production and the concomitant loss of degradation leading to fibrosis will not be discussed in this review, which is focused on the human genetic disorders associated to an altered collagen structure (Table 1) or resulting in excessive degradation of specific collagen elements (Table 2). A general overview of the clinical features associated to these collagen-related disorders is also provided (Table 1 and Table 2).

Since collagens are present throughout the entire body, alterations impairing the quality or quantity of collagen structures can affect any tissue or organ. Since each collagen is generally expressed in several different tissues and it is tightly associated with other ECM elements, alterations result in widely overlapping features that make the diagnosis difficult. Nevertheless, we thereafter propose a tentative classification of the collagen-related disorders according to the major clinical features and affected organs listed in Table 1 and Table 2.

**Skeletal and cartilage abnormalities:** collagen type I is the major ECM component secreted by osteoblasts during bone development. Therefore, alterations in this molecule can give rise to the osteogenesis imperfecta (OI) characterized by bone fragility, as well as the Caffey disease with its infantile episodes of excessive new bone formation (hyperostosis). Also, mutations in *COL2A1* gene result in skeletal abnormalities including the incomplete bone ossification in patients with achondrogenesis type II before birth or the short stature (dwarfism) of patients with Kniest dysplasia. Dwarfism can also be caused by alterations of collagen type IX or X (multiple epiphyseal dysplasia or the Schmid-type metaphyseal chondrodysplasia, respectively) or by mutations in *COL11A1*, *COL11A2* or *COL27A1* genes. Moreover, collagen types II, IX and XI are implicated in the formation and maintenance of the cartilage, thus they are relevant for the joint health and long bone development. Mutations in *COL2A1* gene may result in cartilage alterations characterized by progressive degeneration at the joints (patients with osteoarthritis with mild chondrodysplasia), by hypercellular cartilage with large chondrocytes (Torrance type of platyspondylic lethal skeletal dysplasia) or by a translucent and abnormal gelatinous texture (achondrogenesis type II). Additionally, the presence of fibrous cartilage can be found associated with skeleton defects in patients with fibrochondrogenesis-1 or multiple epiphyseal dysplasia, due to mutations in *COL11A* or *COL9A* gene, respectively.

**Skin alterations:** some of the collagen-related disorders present severe skin alterations. The dystrophic forms of epidermolysis bullosa (EB) with mutations in *COL7A1* or *COL17A1* gene is one of the major forms of EB where patients present a fragile skin, which can shed at the slightest touch. In milder cases blistering may affect the hands, feet, knees and elbows but in severe cases blistering may lead to vision loss, disfigurement and strictures of the gastrointestinal tract. Skin defects are also the main features of patients with the Ehlers-Danlos syndrome (EDS), a genetically heterogeneous disorder with more than nineteen causative genes. Mutations in *COL5A1* or *COL5A2* are responsible for the classical form of EDS. Patients present a soft, velvety skin that is highly stretchy (skin hyperextensibility) and fragile. Affected individuals tend to bruise easily and in some cases, they show atrophic scars. Skin alterations are also found in patients with Bethlem myopathy-1 caused by mutations in *COL6A* genes. Even though muscle dystrophy is the main clinical feature in this group of patients, they present follicular hyperkeratosis on the arms and legs, soft, velvety skin on the hand palms and feet soles, abnormal wound healing that leads to shallow scars.

Finally, alterations of *MMP1* gene expression have been associated to disorders with skin defects: overexpression of *MMP1* in primary dermal fibroblasts of patients with trichothiodystrophy is responsible for collagen type I degradation and altered wound healing features whereas a functional single nucleotide polymorphism in *MMP1* promoter is associated with increased collagen type VII degradation and high severity of recessive dystrophic EB.

**Hearing loss and visual defects:** many of the collagen-related disorders are characterized by sensorineural hearing loss. In particular, all the disorders due to alterations of collagen type XI (see Table 1), the Alport syndrome and the X-linked deafness-6 due to alterations of collagen type IV and the OI and EDS disorders by collagen type I defects may reveal sensorineural hearing loss. Notably, some of these disorders are also associated to visual problems that may include myopia, cataract and in some cases (Stickler syndrome) retinal detachment. More severe vision defects can be observed in patients with the Knobloch syndrome-1 due to alterations of the type XVIII collagen. These patients are affected by high myopia, cataract, dislocated lens, vitreoretinal degeneration and retinal detachment. Finally, alterations of type IV collagen can also result in visual defects as observed in patients with retinal arterial tortuosity, Axenfeld-Rieger anomaly and Small vessel disease of the brain.

**Muscle weakness:** collagen types VI certainly plays a relevant role in skeletal muscle maintenance and regeneration. Alterations of its major elements, the α1 and α2 chains, result in Bethlem myopathy-1, Ullrich congenital muscular dystrophy-1 or the autosomal recessive myosclerosis, all characterized by progressive muscle weakness (hypotonia) and joint stiffness (contractures) with different degree of severity. In the most severe cases weakness of respiratory muscles are reported. Differently, alterations affecting the α3 chain of collagen type VI are found in rare cases with dystonia 27. These patients reveal dystonic action and postural tremor mainly involving the face, neck, bulbar muscles and upper limbs. A severe generalized hypotonia leading to exercise intolerance, feeding difficulties and respiratory insufficiency are present in patients with congenital myasthenic syndrome type 19 due to mutations in *COL13A1* gene. Also, patients with EDS reveal weak muscle tone and hypermobile joints, which can delay the development of motor skills such as sitting, standing and walking. Two cases with OI due to mutations in *SPARC* gene present underdeveloped muscles of the lower extremities, muscle hypotonia and gross motor developmental delay, whereas a single family with congenital fibrosis of extraocular muscles-5 caused by mutations in *COL25A1* gene showed ophthalmoplegia of the extraocular muscles.

Small vessel anomalies and kidney disease: the type IV collagen is the major constituent of the basement membranes. It is a non-fibrillar collagen made of three distinct heterotrimers generated by the products (α chains) of 6 distinct genes. Mutations in *COL4A1* and *COL4A2* result in thickness and damaged vascular basement membranes that affect the straightness of the vessels in patients with susceptibility to intracerebral haemorrhage, hereditary angiopathy or small vessel disease of the brain. Also, the retinal arterial tortuosity derives from mutations in the *COL4A1* gene. Differently, alterations impairing the α3, α4 or α5 chains of collagen type IV result in defects of the glomerular basement membrane that affect kidney functionality. This is observed in patients with Alport syndrome who experience high levels of haematuria and proteinuria due to the progressive loss of kidney activity. Persistent and recurrent haematuria is also observed in the benign form of familial haematuria due to mutations in *COL4A3* or *COL4A4* genes.

Most of the collagen-related disorders affect early childhood health but mild situations can also occur during adulthood. Indeed, the severity of clinical features in patients affected by *Stickler syndrome* varies among individuals and mild cases with late onset have also been reported. Conversely, when collagen alterations result in severe deformations, the survival of the entire organism is compromised as shown by *PPIB* mutations in *type IX osteogenesis imperfecta*. Patients affected by such severe form of OI die during gestation or shortly after birth. It is worthwhile considering that the severity of the disorder relies on several different factors where tissue distribution and function of the affected collagen play leading roles. The OI with its various degree of severity is the result of structural alterations of collagen type I, the most abundant collagen in humans. In particular, mutations inactivating one of *COL1A1* alleles and resulting in reduced levels of an otherwise normal type I collagen are usually responsible for the mild forms of OI whereas dominant negative mutations in *COL1A1* or *COL1A2* genes account for the most severe forms. Notably, among the most common mutations responsible for the severe form of OI are those involving the substitutions of the glycine amino acid in the G-X-Y repeats, essential for the formation of the triple helix. This strongly points to the notion that structural alterations are more detrimental to human health than collagen impoverishment. Similarly, mutations in *COL2A1* gene result in several rare autosomal dominant clinical entities that share skeletal dysplasia, short stature and sensorial defects. The wide range of clinical manifestations were not fully understood but a recent study on over 700 cases (harbouring 415 different mutations) revealed that one-third of the mutations affect the glycine amino acid in the G-X-Y repeats and give rise to the severe *achondrogenesis type II* disease, which is typically identified in utero and may result in embryo death. In contrast, mutations resulting in a premature stop codon or the p.Arg275Cys substitution are responsible for the less severe cases affected by *Stickler syndrome* or *Czech dysplasia* [75]. In summary, the type of mutation, the tissue distribution and the function of the affected collagens all impact on the clinical spectrum of collagen-related disorders.

## 6. Conclusions

The large number of genetic disorders associated to collagen alterations clearly strengthens the relevance of this wide group of proteins, which have been for long time considered inert elements with no other function than maintenance of tissue shape and architecture. The observation that mutations in collagen coding genes result in alterations of relevant developmental processes (skeletal and cartilage development) or defects of tissue homeostasis (skin, sensorineural, visual and muscle alterations) clearly demonstrate a regulatory role for this type of molecules. Therefore, not only the collagens but also the ECM with its broad number of elements and its wide complexity plays a role of primary importance among the mechanisms implicated in embryonic development, normal organ physiology and human health. Moreover, the wide and largely overlapping spectrum of clinical features of collagen, or even ECM-related disorders, makes in some instances the clinical diagnosis and patient management very difficult. A better understanding of the signalling events regulating the expression, functions and dynamic interplay of the various ECM elements will improve our knowledge on the pathogenesis of ECM-related disorders and, in parallel, will provide the tools for the identification of potential therapeutic targets.

## Figures and Tables

**Table 1:** Hereditary disorders resulting from collagen structural alterations ^a.^

Disorder	Collagen Type	Genetic Alteration ^b^	Major Clinical Features ^b^
Ehlers-Danlos syndrome (EDS)	**COL1** **COL3** **COL5**	Mutations in more than a dozen genes have been found to cause EDS (#130000). The classical type 1 and 2 (#130010) result from mutations in either the *COL5A1* (*120215) or *COL5A2* (*120190) gene. Other genes involved are *COL1A1* (*120150), *COL1A2* (*120160), *COL3A1* (*120180). Mutation in *COL1A1* or *COL1A2* lead to the deletion of exon 6 of the mRNA coding the α1 (EDS VIIA, #130060) or the α2 chain (EDS VIIB, #617821) of type I collagen, respectively. Inheritance is autosomal dominant.	EDS is the name associated with at least nine phenotypically characterized clinical entities, which result from different types of mutations in distinct collagen genes and several collagen processing genes. These disorders are biochemically and clinically distinct although they all manifest structural weakness in connective tissue as a result of defects in the structure and function of collagens [76]. Although all types of EDS affect joints and skin, additional features vary by type. Severity ranges from mild to severe. Joint hypermobility occurs with most forms of EDS. Infants with hypermobile joints often have weak muscle tone, which can delay the development of motor skills such as sitting, standing and walking. The loose joints are unstable and prone to dislocation and chronic pain. Many EDS patients have soft, velvety skin that is highly stretchy (skin hyperextensibility) and fragile. Affected individuals tend to bruise easily and in some cases, they show atrophic scars. People with the classical form of EDS experience wounds that split open with little bleeding and leave scars that widen over time to create characteristic “cigarette paper” scars.
		Mutations in the *COL3A1* have been identified in the vascular type of EDS (#130050).	The vascular type can involve serious and potentially life-threatening complications due to unpredictable tearing of blood vessels. This rupture can cause internal bleeding, stroke and shock. The EDS vascular type is also associated with an increased risk of organ rupture, including tearing of the intestine or the uterus (womb) during pregnancy.
		Mutations in *procollagen-lysine, 2-oxoglutarate 5-dioxygenase 1* gene (*PLOD1*, *153454) and *FK506 binding protein 14* (*FKBP14*, *614505) are responsible for the EDS kyphoscoliotic type I and II (EDSKMH, #225400; #614557). Inheritance is autosomal dominant in both cases. PLOD1 catalyses the hydroxylation of lysyl residues in collagen-like peptides, which are critical for the stability of intermolecular crosslinks. FKBP14 acts at the level of the protein folding in the ER, including components of the ECM (COL1, COL3, COL6 and fibronectin).	In addition to the classical symptoms of EDS, patients with EDSKMH I and II are characterised also by progressive kyphoscoliosis with muscle hypotonia from birth, joint laxity, gross motor delay, severe skin hyperelasticity, easy bruising, fragility of sclerae, myopathy and hearing loss [77].
		The Musculocontractural Type I and type II form of EDS (EDSMC1, #601776; EDSMC2, #615539) are caused by recessive loss-of-function mutations in the *carbohydrate sulfotransferase 14* (*CHST14,* *608429) and in the *dermatan sulphate epimerase* (*DSE,* *605942) genes, respectively. The genes encode enzymes involved in the dermatan sulphate (DS) bio-synthesis that is involved in the assembly of collagen fibril. Mutations in both genes lead to the intracellular retention of COL1 and COL3 and a reduced deposition of collagen types I, III, V and VI in the ECM.	EDSMC1 and 2 share most of the clinical features, even though the majority of cases (31) refer to the EDSMC1 and only three cases are reported for the EDSMC2 type [78]. The two syndromes are characterised by progressive kyphoscoliosis, adducted thumbs in infancy or clenched fists and talipes equinovarus, hands with atypically shallow palmar creases and tapering fingers, joint hypermobility, clubfoot, arachnodactyly, elastic skin and poor wound healing. Craniofacial features include brachycephaly, large fontanel, hypertelorism, downslanting palpebral fissures, microcorneae, strabismus, prominent nasolabial folds, short philtrum, thin upper lip, small mouth, high palate and microretrognathia. EDSMC neonates show distal arthrogryposis and muscular hypotonia [78,79].
		The Spondylodysplastic Type 1 (also known as progeroid form of EDS) and 2 forms of EDS (EDSSPD1, #130070; EDSSPD2, #615349) are caused by mutations in the *β-1,4-Galactosyltransferase 7* gene (*B4GALT7*, *604327) and *β-1,3-Galactosyltransferase 6* (*B3GALT6*, *615291), respectively. The genes encode enzymes involved in the production and proper folding of collagen in connective tissue. The Spondylocheiro dysplastic form of EDS (SCD-EDS) results from mutations in the membrane-bound zinc transporter *SLC39A13* (*608735) and has a reliable clinical overlap with EDSSPD1-2. Mutations in *SLC39A13* result in increased Zn^2+^ content inside the endoplasmic reticulum, which inhibits the proper collagen crosslinking and the stability of the collagen triple helix. EDSSPD1 is an autosomal dominant disease whereas EDSSPD2 and SCD-EDS have an autosomal recessive inheritance.	Patients with EDSSPD1-2 showed short stature, muscle hypotonia, radioulnar synostosis and mild to severe intellectual disability (ID). In addition, they present facial dysmorphism, hyperextensible skin, joint hypermobility (JHM), single transverse palmar crease, severe hypermetropia, limb bowing and osteopenia [80].
		The Dermatosparaxis type of EDS (EDSDERMS, #225410) results from mutations in disintegrin and metalloproteinase with thrombospondin motifs (*ADAMTS2*,*604539), the gene encoding the procollagen peptidase that cleavages the N-propeptide of the fibrillar procollagens types I-III and V	The EDSDERMS is characterized by skin that sags and wrinkles. Extra (redundant) folds of skin may be present as affected children get older [81].
Osteogenesis Imperfecta (OI)	**COL1**	Mutations in the *COL1A1* (*120150) and *COL1A2* (*120160) genes are responsible for more than 90% of all cases of OI (#166200). Most of the mutations causative of OI type I affect *COL1A1* gene and result in reduced levels of COL1, whereas those responsible for most of OI types II (#166210), III (#259420) and IV (#166220) cases occur in *COL1A1* or *COL1A2* genes and impair COL1 structure. The inheritance is autosomal dominant.	At least four biochemically and clinically distinguishable forms of OI have been identified associated to defects in COL1. These are named as OI type I (mild), type II (perinatal lethal), type III (deforming) and type IV (mild deforming). A defect in COL1 structure weakens connective tissues, particularly bones. All four forms of OI present reduced levels of COL1 and brittle bones that break easily. Multiple fractures result in bone deformities. Additional symptoms may include blue sclera, short height, loose joints, hearing loss, breathing and teeth problems, cervical artery dissection and aortic dissection [82,83].
		Mutations in *Cartilage associated protein CRTAP,* *605497), *Prolyl 3-hydroxylase* (*P3H1,* *610339) and *Peptidyl-prolyl isomerase B* (*PPIB,* *123841) genes results in OI type VII (#610682), VIII (#610915) and IX (#259440), respectively. *CRTAP* encodes a cartilage-associated protein whereas *P3H1* an enzyme belonging to the collagen propyl hydroxylase family. *PPIB* encodes for a cyclophilins (Cyps) protein that catalyses the *cis*–*trans* isomerisation of peptide bonds. All these proteins are required for proper collagen synthesis, assembly and secretion. In these cases, the inheritance is autosomal recessive.	In addition to the four forms of OI previously described, eleven additional phenotypically related disorders in the OI family exist, all associated with bone fragility and low bone mass. Among the OI associated to collagen alterations, the type VII (mutations in *CRTAP*) is sometimes considered a lethal form with multiple fractures, long bone deformities, scoliosis and short stature [84]. The type VIII form of OI (mutations in *P3H1*) includes severe growth defects, skeletal demineralization, scoliosis, round face and proptosis [85]. The type IX (mutations in *PPIB*) is a very severe form of OI. Embryos dye during pregnancy or few months after birth. Radiographs and an autopsy showed the presence of shortened, bowed and fractured long bones without evident rhizomelia [86].
		The *Serpin family H member 1* (*SERPINH1*, *600943) gene encodes a collagen-binding protein that has chaperone activity in the endoplasmic reticulum. Mutations in *SERPINH1* cause the type X OI (#613848), whose inheritance is autosomal recessive.	Types X OI is a severe deforming form of the disorder characterized by aberrant collagen crosslinking, folding and chaperoning [87].
		Absence of *FK506 binding protein 10* (*FKBP10*, *607063) in recessive type XI OI (#610968) leads to reduced collagen cross-linking and deposition. *FKBP10* encodes a chaperone that contributes to type I procollagen folding. Mutations in this gene affect its secretion.	Clinical hallmarks of OI type XI are congenital contractures. All the others clinical data on the 29 patients with OI type XI (mutations in *FKBP10*) are limited and heterogeneous regarding the age of onset, the number of fractures, the type of affected bones and the severity of the disorder [88].
		The recessive form of OI type XIII (OI13, #614856) is caused by mutations in the *BMP1* (*112264) gene, which is involved in the processing of the C-propeptides of procollagens types I-III and the proteolytic activation of the enzyme lysyl oxidase, necessary for the formation of covalent cross-links in collagen and elastic fibres.	The OI type XIII is characterized by normal teeth, faint blue sclerae, severe growth deficiency, borderline osteoporosis and an average of 10–15 fractures a year affecting both the upper and lower limbs and with severe bone deformity.
		The *Secreted protein acidic and cysteine rich* (*SPARC*,*182120) gene encodes a glycoprotein that binds to COL1 and other ECM proteins. Mutations in this gene are responsible for the type XVII OI (#616507) and seem to result in the over-modification of collagen during triple-helical formation. The inheritance is autosomal recessive.	Two clinical cases have been reported: the first is a girl from North Africa with low bone mineral density (BMD), scoliosis, short stature, mild joint hyperlaxity, weak underdeveloped muscles of the lower extremities, bowing of both humeri and speech delay. The second patient is an Indian girl, who had a left hip dislocation at the age of 10 weeks, muscle hypotonia and gross motor developmental delay. Other features are decreased calf muscle mass, joint hyperlaxity and soft skin [89].
Caffey disease, also called infantile cortical hyperostosis	**COL1**	The *COL1A1* (*120150) variant c.3040C>T (p.Arg836Cys) in exon 41 is the pathogenic variant currently identified in all individuals with Caffey disease (#114000). Inheritance is autosomal dominant but not all people who inherit the mutation develop signs and symptoms. The amino acid change leads to COL1 fibrils that are variable in size and shape.	Caffey disease is characterised by excessive new bone formation (hyperostosis) in early infants. Affected bones may double or triple in width and include jawbone, scapulae, clavicles and the shafts (diaphyses) of long bones in arms and leg. Affected babies are frequently feverish and irritable. They show swelling of joints, pain and redness of affected areas. Usually, there is spontaneous resolution of the inflammatory signs within few months or years. Rare cases of recurrence have also been described [90].
Alpha-2-Deficient Collagen Disease	**COL1**		In 1974 Meigel and co-authors [91] described a 10-year-old son of consanguineous parents, with an apparently ‘new’ connective tissue disorder. The clinical and radiologic abnormalities were reminiscent of both Marfan syndrome and osteogenesis imperfecta. Study of cultured fibroblasts showed a complete failure of synthesis of α-2 chains of collagen.
Spondyloepiphysea l dysplasia congenita (SED)	**COL2**	SED congenita (# 183900) is caused by heterozygous mutation in *COL2A1* gene (*120140) on chromosome 12q13. The inheritance is autosomal dominant.	SED congenita is a chondrodysplasia characterized by short spine, barrel-shaped chest, abnormal epiphyses and flattened vertebral bodies. Skeletal features are manifested at birth and evolve with time. Other features include myopia and/or retinal degeneration with retinal detachment and cleft palate [92].
Stanescu type of spondyloepiphyseal dysplasia (SEDSTN)	**COL2**	SEDSTN (#616583) is caused by heterozygous mutation in *COL2A1* gene (*120140) on chromosome 12q13. The inheritance is autosomal dominant.	Spondyloepiphyseal dysplasia with accumulation of glycoprotein in chondrocytes has been designated the “Stanescu type”. Clinical hallmarks include progressive joint contracture with premature degenerative joint disease, particularly in the knee, hip and finger joints and swollen interphalangeal joints of the hands. The affected individuals are not short, despite the presence of a short trunk. Radiologically, spondylar and epiphyseal abnormalities are quite conspicuous. Other clinical characteristics are generalized platyspondyly, hypoplastic pelvis, epiphyseal flattening with metaphyseal splaying of the long bones and enlarged phalangeal epimetaphyses of the hands [93,94].
Multiple epiphyseal dysplasia with myopia and conductive deafness (EDMMD)	**COL2**	EDMMD (#132450) is caused by heterozygous mutation in *COL2A1* gene (*120140) on chromosome 12q13. The inheritance is autosomal dominant.	EDMMD is characterized by epiphyseal dysplasia associated with progressive myopia, retinal thinning, crenated cataracts, conductive deafness, joint pain, deformity, waddling gait and short stature. In 1978 Beighton and colleagues [95] described an Afrikaner family in South Africa in which the mother, two sons and one daughter had a syndrome of multiple epiphyseal dysplasia, myopia and conductive deafness. The patients had short stature, brachydactyly, genu valgus deformity and dysplasia of the epiphyses. The epiphyses around the knee joint were flattened, the femoral necks were widened and the vertebral bodies were mildly reduced in height and were concave on their upper and lower surfaces.
Achondrogenesis type II (ACG2)	**COL2**	ACG2 (#200610) is caused by mutations in *COL2A1* gene (*120140) on chromosome 12q13. The inheritance is autosomal dominant but somatic and germline mosaicism have also been reported [96].	ACG2 is characterized by severe micromelic dwarfism with small chest and prominent abdomen. Other clinical features include incomplete bone ossification and disorganization of the costochondral junction. The cartilage appears as abnormal gelatinous texture and translucent [75].
Czech dysplasia	**COL2**	Czech dysplasia (#609162) is caused by heterozygous mutations in *COL2A1* gene (*120140) on chromosome 12q13. The inheritance is autosomal dominant.	Czech dysplasia is a skeletal dysplasia characterized by early and progressive onset, shortening of the third and fourth toes caused by metatarsal hypoplasia [97]. Affected individuals have a normal stature but usually complain of severe joint pain before adolescence. Clinical signs are restricted mobility in the lower limb joints and kyphoscoliosis. Skeletal radiographs reveal signs of pseudorheumatoid. Narrow joint spaces and flattened epiphyses platyspondyly with irregular endplates and elongated vertebrae can be observed in the most severe cases. Only five affected families from the Czech Republic have been so far reported [98].
Legg-Calve-Perthes disease (LCPD)	**COL2**	LCPD (#150600) is caused by heterozygous mutation in the *COL2A1* (*120140) gene on chromosome 12q13. The inheritance is autosomal dominant.	LCPD is a form of avascular necrosis of the femoral head (ANFH; #608805) that affects hip development in growing children. It is due to loss of circulation in the femoral head. Radiology does not permit an early diagnosis that depends on the phase of disease progression through ischemia, revascularization, fracture and collapse, repair and remodelling of the bone. LCPD affects more often boys who are usually shorter than their peers [99].
Osteoarthritis with mild chondrodysplasia (OSCDP)	**COL2**	OSCDP (#604864) is caused by heterozygous mutation in *COL2A1* gene (*120140) on chromosome 12q13. The inheritance is autosomal dominant.	OSCDP is a common disease that produces joint pain and stiffness together with radiologic evidence of progressive degeneration of joint cartilage. Several cases have been reported, included family members over various generations [95,100,101]. Major features are primary osteoarthritis associated with mild chondrodysplasia. Over the years the range of motion becomes limited. In about 60% of affected persons, abnormalities of the vertebral bodies consistent with mild chondrodysplasia have been described, including platyspondyly, irregular end plates, herniations within the vertebral bodies (Schmorl nodes) and anterior wedging. Other minor changes include iliac exostoses.
Torrance type of platyspondylic lethal skeletal dysplasia (PLSD-T)	**COL2**	PLSD-T (#151210) can be caused by heterozygous mutation in *COL2A1* gene (*120140) on chromosome 12q13. The disease is transmitted in an autosomal dominant manner. All the patients analysed so far have mutations in the C-propeptide domain of COL2A1, which lead to altered biosynthesis. The phenotype could result from a combination of diminished collagen fibril formation, toxic effects through the accumulation of unfolded collagen chains inside the chondrocytes and/or alteration of a putative signalling function of the C-propeptide.	PLSD-T is a rare skeletal dysplasia characterized by platyspondyly, brachydactyly and metaphyseal changes. Radiology reveals decreased ossification of the skull base, short thin ribs, hypoplastic pelvis with wide sacrosciatic notches and flat acetabular roof, short tubular long bones with ragged metaphyses and bowing of the radius. Histologically, the growth plate appeared relatively normal. The resting cartilage appeared hypercellular with large chondrocytes [102,103]. Though generally lethal in the perinatal period, a few long-term survivors with PLSD-T have been reported [104]. Some patients also present shortening of long bones, degenerative changes in the proximal femora, limited elbow extension, midface hypoplasia, myopia, deafness and mental retardation [105].
Strudwick type of spondyloepimeta-physeal dysplasia (SEMD)	**COL2**	SEMD (#184250) is an autosomal dominant disorder caused by heterozygous mutation in *COL2A1* gene (*120140) on chromosome 12q13.	SEMD clinical features include severe dwarfism, marked pectus carinatum and scoliosis. Cleft palate and retinal detachment are frequently associated. Distinctive radiographic feature is irregular sclerotic changes, described as “dappled” in the metaphyses of the long bones that are caused by alternating zones of osteopenia and osteosclerosis [106].
Spondyloperipheral dysplasia	**COL2**	Spondyloperipheral dysplasia (#271700) is autosomal dominant disorder caused by heterozygous mutation in *COL2A1* gene (*120140) on chromosome 12q13.	The disorder is a skeletal dysplasia with platyspondyly and brachydactyly E-like changes (short meta-carpals and metatarsals, short distal phalanges in the hand and feet) [107].
Stickler syndrome (STL)	**COL2, COL9** **COL11**	Pathogenic variants in one of six genes (*COL2A1, COL11A1, COL11A2, COL9A1, COL9A2* and *COL9A3*) can be associated with Stickler syndrome. STL is inherited in autosomal dominant manner when mutated in *COL2A1, COL11A1* or *COL11A2*, in autosomal recessive manner when mutated in *COL9A1*, *COL9A2*, *or COL9A3*.	STL is a genetically heterogeneous connective tissue disorder characterized by myopia, cataract and retinal detachment, conductive and sensorineural hearing loss. Additional findings may include mid–facial underdevelopment and cleft palate, mild spondyloepiphyseal dysplasia and/or precocious arthritis. Variable phenotypic expression occurs within and among families. Interfamilial variability is partially explained by locus and allelic heterogeneity [108].
Stickler syndrome type I (STL1)		STL1 (#108300), also called the membranous vitreous type, is caused by heterozygous mutation in *COL2A1* gene (*120140) on chromosome 12q13.	STL1 patients usually display a congenital vitreous abnormality consisting of a vestigial gel in the retrolental space, bounded by a highly folded membrane. Most affected individuals are at high risk for retinal detachment. Systemic features typically seen in STL1 are premature osteoarthritis, cleft palate, hearing impairment and craniofacial abnormalities [109].
Stickler syndrome type II (STL2)		STL2 (#604841), sometimes called the beaded vitreous type, is caused by heterozygous mutation in *COL11A1* gene (*120280) on chromosome 1p21.	Patients affected by STL2 are myopic, rarely with paravascular lattice retinopathy. They frequently present cataract or are aphakic or pseudophakic. Retinal detachment, either mono- or bi-lateral may appear in the 3rd decade. Moreover, *COL11A1* mutations are associated by early-onset hearing loss [110].
Stickler syndrome type III (STL3)		STL3 (#184840) or “nonocular Stickler syndrome” has been recently reclassified as form of otospondylomegaepiphyseal dysplasia or Weissenbacher-Zweymuller syndrome (OSMEDA or WZS). It is caused by heterozygous mutations in *COL11A2* gene (*120290) on 6p21 chromosome.	Patients affected by STL3 have typical facial features, including midface hypoplasia combined with hearing impairment. No ocular abnormalities are reported. They present relatively short extremities with abnormally large knees and elbows but normal total body length. Diagnostic radiologic findings are enlarged epiphyses combined with moderate platyspondyly, mainly in the lower thoracic region [111].
Stickler syndrome type IV (STL4)		STL4 (#614134) is caused by homozygous mutation in *COL9A1* gene (*120210) on chromosome 6q13.	Individuals affected by STL4 have moderate-to-severe sensorineural hearing loss, moderate-to-high myopia with vitreoretinopathy, cataracts and epiphyseal dysplasia [112]. The vitreous abnormality resembles an aged vitreous rather than the typical membranous, beaded or non-fibrillar type.
Stickler syndrome type V (STL5)		STL5 (#614284) is caused by homozygous mutation in *COL9A2* gene (*120260) on chromosome 1p34.	One family with STL5 has been reported. Major clinical findings are high myopia, vitreoretinal degeneration, retinal detachment, hearing loss and short stature. None of the family members was known to have cleft palate and, although there was short stature in childhood, normal height was found in adults [108].
Stickler syndrome atypical		The atypical form of STL (#609508) with predominantly ocular findings is caused by mutation in *COL2A1* gene (*120140). The inheritance is autosomal dominant.	Patients display high myopia and retinal detachment. Systemic features of premature osteoarthritis, cleft palate, hearing impairment and craniofacial abnormalities are very mild or absent [113].
Familial avascular necrosis of the femoral head-1 (ANFH1)	**COL2**	ANFH1 (#608805) is an autosomal dominant disorder caused by heterozygous mutation in *COL2A1* gene (*120140) on chromosome 12q13.	ANFH1 is a debilitating disease affecting young adults between 35 and 55 years of age. The disorder is characterized by progressive pain in the groin, mechanical failure of the subchondral bone and degeneration of the hip joint. Nearly half of patients require hip replacement before 40 years of age [114].
Kniest dysplasia	**COL2**	Kniest dysplasia (#156550) is caused by mutations in *COL2A1* gene (*120140). The inheritance is autosomal dominant.	Patients have short stature, flat facial profile, high myopia, risk of retinal detachment, cleft palate, deafness, high risk of severe degenerative joint disease and odontoid hypoplasia leading to risk of atalantoaxial instability and paralysis. Other features include neonatal respiratory distress, infantile hypotonia, abnormal oval-shaped vertebra at birth and later platyspondyly, shortened, “dumbbellshaped” long bones, with splaying of the epiphyses and metaphyses [115].
Alport syndrome	**COL4**	Alport syndrome is a clinically and genetically heterogeneous nephropathy. Approximately 80% of cases are transmitted as an X-linked semi-dominant condition due to *COL4A5* mutations. 20% of cases are autosomal recessive due to mutation in either *COL4A3* or *COL4A4*. Same families with autosomal-dominant Alport syndrome have been reported, either caused by *COL4A3* or *COL4A4* mutations.	Alport syndrome is characterized by progressive nephritis associated with hearing loss and sometime ocular lesions. Patients experience progressive loss of kidney function. The majority of affected individuals have blood (haematuria) and high levels of proteins (proteinuria) in their urine, which indicate impaired kidney function. Many patients also develop hypertension and at end-stage renal disease. Ocular anomalies are frequent in Alport syndrome and they can precede proteinuria in 40% of patients. Anterior lenticonus, abnormal coloration of the retina, lens rupture, cataracts and corneal erosions can be found [116]. Pregnancy of patients with Alport syndrome is very challenging and often complicated by deterioration of renal function, preeclampsia, severe placental dysfunction and sometime acute renal failure. Preterm delivery is frequent [117].
Alport syndrome autosomal dominant		The autosomal dominant form of Alport syndrome (#104200) is caused by heterozygous mutation in *COL4A3* gene (*120070).	
Alport syndrome X-LINKED (ATS)		ATS (#301050) is caused by mutations in *COL4A5* (*303630) gene. The inheritance is dominant.	ATS males are more severely affected than females. Men have a 90% chance of developing end-stage kidney disease by age 40. Patients with large deletions or nonsense mutations have significantly earlier onset than those with missense mutations. The majority (95.5%) of women with *COL4A5* mutations develop microscopic haematuria [118].
Leiomyomatosis, diffuse, with Alport syndrome (DL-ATS)		DL-ATS (#308940) is caused by large deletions involving *COL4A5* (*303630) and *COL4A6* (*303631) genes. Likely an X-linked semi-dominant inheritance.	DL-ATS reveals the Alport syndrome features associated with diffuse leiomyomatosis [119].
Alport syndrome autosomal recessive		This form of Alport syndrome (#203780) is caused by mutations in *COL4A3* (*120070) or *COL4A4* (*120131) gene.	Autosomal recessive Alport syndrome presents as gross proteinuria in childhood and progression to end-stage kidney disease often before the fourth decade [120].
Autosomal dominant mental retardation-34 (MRD34)	**COL4**	MRD34 (#616351) is caused by heterozygous mutation in *COL4A3BP* (*604677) gene on chromosome 5q13. The inheritance is autosomal dominant.	Patients with MRD34 present unremarkable perinatal history and delivery with a normal birth weight. Neonatal feeding difficulties may occur. Psychomotor development is delayed and speech skills limited. Auto-mutilation behaviour and anxiety are observed. Normal growth parameters and no evident dysmorphism are recorded in adults [121,122].
Retinal arterial tortuosity (RATOR)	**COL4**	RATOR (#180000) is caused by heterozygous mutation in *COL4A1* gene (*120130) on chromosome 13q34. The inheritance is autosomal dominant. One single family with approximately 20 familial cases has been reported so far.	RATOR is an uncommon condition characterized by marked tortuosity of second- and third-order retinal arteries with normal first-order arteries and venous system. Typically, the vascular tortuosity is predominantly located at the macular and peripapillary area and develops during childhood or early adulthood. Although the disease may be asymptomatic, most patients complain of variable degrees of transient vision loss due to retinal haemorrhage following physical exertion or minor trauma. Involvement of non-ocular vascular beds has not been demonstrated in most cases but occasionally other associated vascular abnormalities have been recorded, including malformations in the Kieselbach nasal septum, spinal cord vascular mass, telangiectasis of bulbar conjunctiva and internal carotid artery aneurysm [123].
Hereditary angiopathy with nephropathy, aneurysms and muscle cramps (HANAC)	**COL4**	HANAC (#611773) is caused by heterozygous mutation in *COL4A1* gene (*120130) on chromosome 13q34. The inheritance is autosomal dominant.	HANAC syndrome is characterized by angiopathy that affects several parts of the body. Patients present kidney alterations consisting of multiple renal cysts and sometimes haematuria. The brain is only mildly affected and intracranial aneurysms causing haemorrhagic stroke can occur. Leukoencephalopathy is found in about half of affected individuals whereas muscle cramps are experienced by most of patients in early childhood. In addition, patients may manifest eye problems, like arterial retinal tortuosity, cataract and abnormality called Axenfeld-Rieger anomaly [124].
Small vessel disease of the brain with or without ocular anomalies (BSVD)	**COL4**	BSVD (#607595) is caused by heterozygous mutation in *COL4A1* gene (*120130) on chromosome 13q34. The inheritance is autosomal dominant.	BSVD is characterized by a wide spectrum of symptoms of varying severity including porencephaly variably associated with eye defects (retinal arterial tortuosity, Axenfeld-Rieger anomaly, cataract) and systemic findings such as kidney involvement, muscle cramps, cerebral aneurysms, Raynaud phenomenon, cardiac arrhythmia and haemolytic anaemia. Stroke is often the first symptom and is usually caused by haemorrhagic rather than ischemic stroke. Patients also have leukoencephalopathy and may experience infantile hemiparesis, seizures and migraine headaches accompanied by visual auras [125].
Porencephaly	**COL4**	Porencephaly is an autosomal dominant disorder characterize by mutations in *COL4A1* (*120130) or *COL4A2* (*120090) genes on chromosome 13q34.	It is a neurological disorder characterized by fluid-filled cysts or cavities in the brain and is thought to result from disturbed vascular supply leading to cerebral degeneration. Affected individuals have delayed growth and development, hypotonia, spastic hemiplegia, seizures, migraine headaches, speech problems and intellectual disability with variable severity [126].
Porencephaly-1 (POREN1)		POREN1 (#175780) is caused by mutations in *COL4A1* gene.	POREN1 is more common. It is usually unilateral and results from destructive lesions.
Porencephaly-2 (POREN2)		POREN2 (#614483) is caused by mutations in *COL4A2* gene.	POREN2 is usually symmetrical and results from developmental malformation.
Schizencephaly	**COL4**	Some patients with schizencephaly (#269160) have mutations in *COL4A1* (*120130) gene.	Schizencephaly is a very rare cortical malformation that results in grey matter line clefts impacting one or both sides of the brain. Two types of schizencephaly have been described, depending on the size of the area involved and on the separation of the cleft lips. The clinical picture is mainly based on the presence of motor deficits and mental retardation but the severity of the symptoms varies depending on the size and location of the clefts and on the presence of associated cerebral malformations. Patients with type I are almost normal, they may have seizures or motor impairment. Type II is associated with mental retardation, seizures, hypotonia, spasticity, inability to walk or speak and blindness [127].
Susceptibility to intracerebral haemorrhage (ICH)	**COL4**	ICH (#614519) may be due to mutations in *COL4A2* (*120090) or *COL4A1* (*120130) genes on chromosome 13q34. The inheritance is autosomal dominant.	Few patients with adult-onset haemorrhagic stroke have been reported. The mutated vascular collagen diminishes the tensile strength of vessels and increases their fragility, which can lead to haemorrhage [128].
X-linked deafness-6 (DFNX6)	**COL4**	DFNX6 (#300914) is caused by mutation in *COL4A6* gene (*303631) on chromosome Xq22. One family has been reported so far.	The symptoms vary in male and female patients affected by this disorder. The severe bilateral sensorineural hearing loss apparent in infancy affects only males, who present bilateral malformation of the cochlea with incomplete separation from the internal auditory canal. Language skills in these patients are severely restricted. Female patients develop mild to moderate hearing impairment in the third/fourth decades of life and rarely hearing loss in the first decade of life [129].
Benign familial haematuria (BFH)	**COL4**	BFH (#141200) are caused by mutations in *COL4A3* (*120070) or *COL4A4* (*120131) gene, both of which map on chromosome 2q36. The inheritance is autosomal dominant.	BFH is characterized by the presence of persistent or recurrent haematuria, usually detected in childhood. Haematuria remains isolated and never results in end-stage renal disease. Diffuse attenuation of the glomerular basement membrane is usually considered the hallmark of the condition but it is not specific [130].
Bethlem myopathy-1 (BTHLM1)	**COL6**	BTHLM1 (#158810) is caused by mutations in *COL6A1* (*120220), *COL6A2* (*120240) or *COL6A3* (*120250) genes, giving rise to the altered or even lack of type VI collagen. Both recessive and dominant mutations have been reported.	The disease is characterized by progressive muscle weakness and joint stiffness (contractures). The features can appear at any age, in some cases before birth (decreased foetal movements) in other cases during infancy with joint laxity (loose joints) and hypotonia (weak muscle tone). Later, during childhood, patients develop contractures in their fingers, wrists, elbows and ankles. When adult, they may develop weakness in respiratory muscles, which result in breathing difficulty. The mild form may also reveal skin abnormalities, including follicular hyperkeratosis on the arms and legs; soft, velvety skin on the hand palms and feet soles; abnormal wound healing resulting in shallow scars [131].
Ullrich congenital muscular dystrophy-1 (UCMD1)	**COL6**	UCMD1 (#254090) is caused by mutations in *COL6A1* (*120220), *COL6A2* (*120240) or *COL6A3* (*120250) genes, giving rise to the altered or even lack of type VI collagen. The disease is transmitted in an autosomal recessive manner and only in rare cases in a dominant pattern.	Patients suffer from a severe muscle weakness beginning soon after birth. Some affected individuals are never able to walk and others can walk only with support. Several lose ambulation ability in adolescence. Progressive scoliosis and deterioration of respiratory function is a typical feature. Some patients need continuous mechanical ventilation to help them breathing. Affected individuals develop contractures in their neck, hips and knees, which further impair movement. There may be joint laxity in patient fingers, wrists, toes, ankles and other joints. As in BTHLM1, some people with UCMD1 have follicular hyperkeratosis [132].
Autosomal recessive myosclerosis	**COL6**	The autosomal recessive myosclerosis (#255600) has an autosomal recessive inheritance and is caused by mutations in *COL6A2* gene (*120240). One family has been reported so far.	The disorder is characterized by chronic inflammation of skeletal muscle with hyperplasia of the interstitial connective tissue. The clinical symptoms include slender muscles with “woody” consistency and restriction of movement of many joints because of muscle contractures. Muscles are thin and may result sclerotic on palpation. The few patients so far described showed difficulty in running and climbing stairs and had Achilles tendon contractures during early childhood. Skeletal muscle biopsies showed a myopathic pattern with fibrosis, proliferation of endomysial and perimysial connective tissue, variation of myofibre diameter. Increased serum creatine kinase was also found [133].
Dystonia 27 (DYT27)	**COL6**	DYT27 (#616411) is caused by compound heterozygous mutations in *COL6A3* gene (*120250) on chromosome 2q37. It is an autosomal recessive disorder.	Neurological disorder characterized by the onset of segmental isolated dystonia involving the face, neck, bulbar muscles and upper limbs in the first two decades of life. Few cases have been reported and the symptoms included dystonic action and postural tremor, writer’s cramp, oromandibular and laryngeal dystonia [134].
The dystrophic forms of epidermolysis bullosa (DEB)	**COL7** **COL17**	The autosomal dominant form of epidermolysis bullosa dystrophica (DDEB, #131750) is caused by heterozygous mutations in *COL7A1* gene (*120120) on chromosome 3p21. The autosomal recessive dystrophic form of epidermolysis bullosa (RDEB, #226600) and the RDEB localized variant (#226650) are caused by homozygous or compound heterozygous mutations in *COL17A1* gene (*113811).	Epidermolysis bullosa (EB) is a term referring to a family of disorders that are associated with excessive blistering in response to mechanical injury or trauma. Microscopic examination of the skin shows cleavage below the basement membrane within the papillary dermis. The signs and symptoms of this condition vary widely among affected individuals. In mild cases, blistering may primarily affect the hands, feet, knees and elbows. Severe cases involve widespread blistering leading to vision loss, disfigurement and other serious medical problems such as strictures of the gastrointestinal tract leading to poor nutrition. Patients show an increased risk of developing aggressive squamous cell carcinoma. Kids with EB are often defined “butterfly wing” children because of their extremely fragile skin, which can shed at the slightest touch. DEB is one of the major forms of EB. DDEB and RDEB are also known as Cockayne-Touraine disease and Hallopeau-Siemens disease, respectively [135]. Variations in severity are observed among the different forms of RDEB. Notably, a functional SNP in *MMP1* (*120353) promoter is associated with high severity in RDEB. Since COL7 is degraded by MMP1, an imbalance between COL7 synthesis and degradation could worsen the RDEB phenotype [136].
Nonsyndromic congenital nail disorder-8 (NDNC8)	**COL7**	NDNC8 (#607523) is caused by heterozygous mutations in *COL7A1* gene (*120120) on chromosome 3p21.1. The disorder is inherited in an autosomal dominant manner.	This form of isolated toenail dystrophy has been found in few Japanese families in which other members had the autosomal recessive dystrophic epidermolysis bullosa (RDEB, #226600) or the transient bullous dermolysis of the newborn (#131705), the features of which include dystrophic nails. The nail plates of the toes were buried in the nail bed and the free edge of the toenail was deformed and narrow [137].
Fuchs endothelial corneal dystrophy-1 (FECD1)	**COL8**	FECD1 (#136800) is caused by heterozygous mutations in *COL8A2* gene (*120252) on chromosome 1p34. It is an autosomal dominant disorder.	FECD is a progressive, bilateral condition leading to reduced vision quality due to dysfunction of the corneal endothelial cells, a thin layer of cells in the back of the cornea that regulates the amount of fluid inside the cornea. FECD occurs when the endothelial cells die and the cornea becomes swollen with too much fluid. Corneal endothelial cells continue to die over time, resulting in further vision problems. Ultrastructural features include loss and attenuation of endothelial cells with thickening and excrescences (guttae) of the underlying basement membrane that are the clinical hallmark of FECD and that worsen with disease progression. As the endothelial layer develops confluent guttae in the central cornea, the cornea becomes dehydrated and clear [138]. In the USA about 5% of the over 40 population is affected by FECD and some early-onset cases are due to *COL8A2* mutations.
Posterior polymorphous corneal dystrophy (PPCD2)	**COL8**	A single family with PPCD2 (#609140) caused by heterozygous missense mutation in *COL8A2* gene (*120252) has been described. Another family with one PPCD2 patient and few FECD cases, due to heterozygous missense mutation in *COL8A2*, has been described [139].	Father and daughter with PPCD2 have been reported. The patients show a bilateral penetrating keratoplasty at the age of twenties (daughter) and fifties (father). The authors suggested that the underlying pathogenesis of FECD and PPCD2 may be related to disturbance of the role of COL8 in influencing the terminal differentiation of the neural crest-derived corneal endothelial cell [140].
Multiple epiphyseal dysplasia (EDM)	**COL9**	There are two types of EDM, which can be distinguished by their pattern of inheritance, the dominant and recessive types. EDM caused by mutations affecting collagen structures have an autosomal dominant transmission. Mutations in *COL9A1*, *COL9A2* or *COL9A3* genes are found in less than 5% of individuals with dominant EDM.	EDM is a clinically and genetically heterogeneous skeletal disorder, which is characterized by joint pain and stiffness, mild short stature and degenerative joint features. Both cartilage and bone development are affected, mainly at the ends of the long bones in the arms and legs (epiphyses). It has been suggested that mutations in *COL9A1*, *COL9A2* or *COL9A3* genes may cause COL9 to accumulate inside the cell or interact abnormally with other cartilage components.
Multiple epiphyseal dysplasia-2 (EDM2)		EDM2 (#600204) is caused by heterozygous mutation in *COL9A2* gene (*120260) on chromosome 1p34.	EDM2 onset is usually in childhood, around 3-4 years of age and clinical variability is observed even within the same family [141].
Multiple epiphyseal dysplasia-3 (EDM3)		EDM3 (#600969) is caused by heterozygous mutation in *COL9A3* gene (*120270).	EDM3 patients show early-onset short stature, waddling gait and pain/stiffness in the knees. Few patients experience involvement of elbow, wrist or ankle [142].
Multiple epiphyseal dysplasia-6 (EDM6)		EDM6 (#614135) is caused by heterozygous mutation in *COL9A1* gene (*120210) on chromosome 6p13. One single family has been reported.	A 30-year-old proband was reported with knee pains and difficulty walking since 10 years of age. Radiographs showed early osteoarthritis of one knee, Schmorl nodes, endplate irregularities, anterior osteophytes in the thoracolumbar vertebrae and normal hips. The mother had the same mutation but she did not reveal any symptom before age 45 years [143]
Schmid-type metaphyseal chondrodysplasia (MCDS)	**COL10**	MCDS (#156500) is caused by heterozygous mutation in *COL10A1* (*120110) gene on chromosome 6q22. MCDS is transmitted as an autosomal dominant trait.	MCDS is a rare genetic disorder characterized by short stature, short arms and legs (short-limbed dwarfism) and bowing of the long bones. Radiographic features include widening and irregularity of the growth plates, especially in the distal and proximal femora. These defects give rise to unusual “waddling” walk (gait) [144].
Marshall syndrome (MRSHS)	**COL11**	MRSHS (#154780) is an autosomal dominant genetic disorder caused by mutations in *COL11A1* gene (*120280) on chromosome 1p21.	Patients have a distinctive flat midface with a flattened nasal bridge (saddle nose), nostrils that turn upward, widely spaced eyes, high myopia, cataracts and sensorineural hearing loss. Other symptoms include crossed eyes (esotropia), retinal detachment, glaucoma, protruding upper incisors (teeth) and a small or missing nasal bone [145].
Fibrochondrogenesis-1 (FBCG1)	**COL11**	FBCG1 (#228520) is a severe, autosomal recessive disorder caused by mutations in *COL11A1* gene (*120280) on chromosome 1p21.	FBCG1 and FBCG2 are short-limbed skeletal dysplasia frequently lethal. The disorder is named for the disorganized cartilage growth plate in which chondrocytes have a fibroblastic appearance and the presence of fibrous cartilage extracellular matrix. Patients are characterized by short stature (dwarfism) and skeletal abnormalities. Affected individuals have shortened long bones in the arms and legs that are unusually wide at the ends (described as dumbbell-shaped). Hands and feet are relatively normal. Vertebrae are flattened (platyspondyly) and have a characteristic pinched or pear shape that is noticeable on x-rays. Ribs are typically short and wide and have metaphyseal cupping at both ends. Affected infants have a very narrow chest, which prevents the lungs from developing normally. Most infants are stillborn or die shortly after birth from respiratory failure. Some affected individuals have lived into childhood. Affected individuals who survive the neonatal period have high myopia, mild to moderate hearing loss and severe skeletal dysplasia [146].
Fibrochondrogenesis-2 (FBCG2)		FBCG2 (#614524) can have an autosomal recessive or dominant inheritance due to mutations in *COL11A2* gene (*120290) on chromosome 6p21.3.	
Autosomal dominant deafness-13 (DFNA13)	**COL11**	DFNA13 (#601868) is an autosomal dominant disorder caused by heterozygous mutation in *COL11A2* gene (*120290) on chromosome 6p21.	A single family has been described, characterized by a dominant nonsyndromic postlingual hearing loss. The affected individuals experienced progressive hearing loss beginning in the second to fourth decades [147].
Otospondylo-megaepiphyseal dysplasia, autosomal dominant (OSMEDA)	**COL11**	The autosomal dominant OSMEDA (#184840), also known as Weissenbacher-Zweymuller syndrome (WZS), is caused by heterozygous mutation in *COL11A2* gene (*120290) on chromosome 6p21. The disorder has an autosomal dominant transmission.	OSMED is characterized by skeletal abnormalities, distinctive facial features and severe hearing loss. The term “otospondylomegaepiphyseal” refers to the parts of the body that are affected: ears (oto-), bones of the spine (spondylo-) and the ends (epiphyses) of long bones in the arms and legs. The disorder is characterized by sensorineural hearing loss, relatively short extremities with abnormally large knees and elbows (enlarged epiphyses), vertebral body anomalies and characteristic facies. The diagnostic radiologic findings are enlarged epiphyses combined with moderate platyspondyly, mainly in the lower thoracic region. No ocular abnormalities are reported. Patients have typical facial features, including midface hypoplasia [148].
Autosomal recessive (OSMEDB)		The autosomal recessive OSMEDB (#215150) is also caused by mutation in the *COL11A2* gene.	
Congenital myasthenic syndrome type 19 (CMS19)	**COL13**	CMS19 (#616720) is an autosomal recessive disorder resulting from mutations in *COL13A1* gene (*120350) on chromosome 10q22.	The congenital myasthenic syndromes (CMSs) are a heterogeneous group of inherited disorders resulting from impaired neuromuscular transmission and caused by mutations in genes involved in the formation or integrity of neuromuscular junctions (NMJs). CMS19 result in generalized muscle weakness, exercise intolerance and respiratory insufficiency. Patients present hypotonia, feeding difficulties and respiratory problems soon after birth. The severity of the weakness and disease course is variable [149].
Epithelial recurrent erosion dystrophy (ERED)	**COL17**	ERED (#122400) is caused by heterozygous mutation in *COL17A1* gene (*113811) on chromosome 10q24. The disorder is transmitted as an autosomal dominant trait.	ERED is characterized by bilaterally painful recurrent corneal erosions. Erosions often are precipitated by relatively minor trauma and are often difficult to treat, lasting for up to a week. Fortunately, the erosions become less frequent as patients age and may cease altogether by the fifth decade of life. The onset is in the first decade of life (even in the first year of life) often with some subepithelial haze or blebs while denser centrally located opacities develop with time. Small grey anterior stromal flecks associated with larger focal grey-white disc-shaped, circular or wreath-like lesions with central clarity, in the Bowman layer and immediately subjacent anterior stroma, varying from 0.2 to 1.5 mm in diameter, may be diagnostic of ERED [150].
Knobloch syndrome-1 (KNO1)	**COL18**	KNO1 (#267750) is a hereditary autosomal recessive disorder caused by mutations in *COL18A1* gene (*120328) on chromosome 21q22.3.	KNO1 is primarily characterized by severe vision problems and skull defects. Eye abnormalities include high myopia, cataracts, dislocated lens, vitreoretinal degeneration and retinal detachment. Skull defects range from occipital encephalocele to occult cutis aplasia [151].
Congenital fibrosis of extraocular muscles-5 (CFEOM5)	**COL25**	CFEOM5 (#616219) has an autosomal recessive inheritance and is caused by mutations in *COL25A1* gene (*610004) on chromosome 4q25. A single family had been reported so far.	CFEOM include several different inherited strabismus syndromes characterized by congenital restrictive ophthalmoplegia affecting extraocular muscles innervated by the oculomotor and/or trochlear nerves. CFEOM5 has been reported in a single family with 3 sibs showing a congenital cranial dysinnervation affecting the ocular muscles. The patients had variable abnormal ocular motility without other systemic defects. Two sibs showed congenital ptosis with levator palpebrae muscle dysinnervation of one or both orbits. The levator palpebrae muscle was normally innervated by cranial nerve III (oculomotor nerve). The third sib had no ptosis but showed bilateral Duane retraction syndrome, exotropic in the right eye and esotropic in the left [152].
Steel syndrome (STLS)	**COL27**	STLS (#615155) displays an autosomal recessive inheritance due to mutations in *COL27A1* gene (*608461) on 9q32 chromosome. Few cases have been reported who belong to the same family.	Patients affected by STLS present a characteristic facies, dislocated hips and radial heads, carpal coalition (fusion of carpal bones), short stature, scoliosis and cervical spine anomalies. The dislocated hips are resistant to surgical intervention [153].

^a^ Only hereditary disorders resulting in structural collagen alterations are listed; ^b^ Information mainly based on OMIM, the Online Mendelian Index in Man at http://www.ncbi.nlm.nih.gov/entrez/query.fcgi?db=OMIM. Grey and white rows are used to distinguish the different disorders. Collagen types are in bold. Abbreviations: COL: collagen. The acronyms of the pathologies are all specified inside the Table.

**Table 2:** Hereditary disorders associated to reduced synthesis or excessive degradation of specific collagen types.

Disorder	Genetic Alteration	Link with the Disorder	Major Clinical Features ^a^
Autosomal recessive dystrophic epidermolysis bullosa (RDEB)	A defect in collagenase *MMP1* (*120353) has been implicated in RDEB (#226600). An association between disease severity and specific SNP in *MMP1* gene (*120353) was found in three affected members of one family and in a cohort of 31 unrelated French RDEB patients [136]. The SNP results in increased transcript and active MMP1 protein levels.	COL7 is susceptible to degradation by the collagenase matrix metalloproteinases-1 (MMP1). An imbalance between COL7 synthesis and degradation could conceivably worsen the RDEB phenotype.	Patients with RDEB present generalized blisters at birth that result in extensive scarring and pseudosyndactyly. After birth, extensive blisters may affect the mucous membranes particularly the oral cavity, oesophagus and anal canal. Caused by chronic blood loss, inflammation, infection and poor nutrition, patients develop anaemia, failure to thrive, delayed puberty and osteoporosis. Patients usually do not survive more than 30 years due to severe renal complications or aggressive squamous cell carcinoma arising in the areas of repeated scarring [154].
Aneurysm, abdominal aortic (AAA)	Mapped loci for AAA (#100070) include *AAA1* (*100070) on chromosome 19q13, *AAA2* (*609782) on chromosome 4q31, *AAA3* (*611891) on chromosome 9p21 and AAA4 (*614375) on chromosome 12q13. Inheritance is autosomal dominant.	Several studies pointed to a role of MMPs in the end-stage of AAA. MMPs are enzymes capable of degrading connective tissue that may affect arterial walls by degrading collagens and other ECM components. Polymorphisms in *MMP2*, *MMP3*, *MMP9* and *MMP13* genes result in increased protein levels significantly associated to AAA risk.	AAA is characterized by chronic inflammation and ECM degradation of the aortic wall. The main symptoms of this condition are dysphasia, frontotemporal cerebral atrophy and frontotemporal dementia, speech disorder, memory impairment [155].
Trichothiodystrophy 1, photosensitive form (TTD1)	TTD1 (#601675) is caused by homozygous or compound heterozygous mutation in the *ERCC2/XPD* gene (*126340) on chromosome 19q13. The gene encodes a helicase subunit of the transcription/repair factor TFIIH. The inheritance is autosomal recessive.	A reduced expression of *COL6A1* (*120220), an abundant collagen of skin and connective tissue, has been shown in the skin of TTD patients with mutations in the *ERCC2/XPD* gene [156]. It has been shown that specific transcription deregulations in the cells of TTD patients with mutations in the *ERCC2/XPD* gene result in the overexpression of *MMP1* gene. This event leads to hyper-secretion of active MMP1 enzyme and degradation of collagen type I in the dermis of TTD patient skin [157].	TTD is characterized by hair abnormalities, physical and mental retardation, ichthyosis, signs of premature aging and cutaneous photosensitivity. The clinical spectrum of TTD varies widely from patients with only brittle, fragile hair to patients with the most severe neuroectodermal symptoms. TTD patients present sulphur-deficient brittle hair with a diagnostic alternating light and dark banding pattern (called ‘tiger’ tail banding) under polarizing microscopy. Common additional clinical features include collodion baby, characteristic facies, ocular abnormalities, short stature, decreased fertility and recurrent infections. TTD patients present a 20-fold higher mortality compared to the US general population [158,159].
Atopic dermatitis (ATOD)	ATOD (#603165) is caused by the presence of a specific SNP (rs4688761) in *COL29A1* gene (*611916), which encodes a novel epidermal collagen. The gene is on chromosome 3q22.1. Inheritance is autosomal dominant.	*COL29A1* shows a specific gene expression pattern with the highest transcript levels in skin, lung and gastrointestinal tract, which are the major sites of allergic disease manifestation. Lack of *COL29A1* expression in the outer layers of the epidermis of ATOD patients points to a role of collagen XXIX in epidermal integrity, whose breakdown is a clinical hallmark of AD [160].	ATOD is a chronic inflammatory skin disease characterized by intensely itchy skin lesions. The onset of disease is typically observed during the first two years of life [161]. The hallmarks of atopic dermatitis are a chronic relapsing form of skin inflammation, a disturbance of epidermal barrier function that culminates in dry skin and IgE-mediated sensitization to food and environmental allergens.
Bruck syndrome (BRKS)	BRKS is a very rare autosomal recessive syndrome. Two forms are found: BRKS1 (#259450) is caused by mutations in *FKBP10* (*607067) gene whereas BRKS2 (#609220) by mutations in *PLOD2* (*601865) gene.		BRKS is characterized by bone fragility associated with congenital joint contractures. Patients commonly show short stature, skull wormian bones and kyphoscoliosis. Most cases had normal teeth, white sclera, normal cognitive functions and normal hearing. A few cases had dysmorphic features including triangular face and brachycephaly [162].
Bruck syndrome 1 (BRKS1)	BRKS1 (#259450) is caused by homozygous mutations in *FKBP10* gene (*607063) on chromosome 17q21 resulting in FKBP65 loss of function. Inheritance is autosomal recessive.	Mutations in *FKBP10* result in delay of type 1 procollagen secretion, incomplete stabilization of collagen trimer, reduced hydroxylation of the telopeptide lysyl residue (involved in intermolecular collagen cross-linking).	BRKS1 patients have short stature, high incidence of joint contractures, frequent fractures and scoliosis.
Bruck syndrome 2 (BRKS2)	BRKS2 (#609220) is caused by homozygous mutation in *PLOD2* gene (*601865) on chromosome 3q24. Inheritance is autosomal recessive.	*PLOD2* encodes the telopeptide lysyl hydroxylase required for the triple-helical cross-linking of collagen molecules. Mutations in this gene affect the instalment and secretion of collagen fibres from osteoblasts [163].	No phenotypic differences between BRKS1 and BRKS2 have been reported.
Ehlers-Danlos syndrome (EDS) subtypes	The EDS subtypes are due to mutations in several genes, including *PLOD1*, *FKBP14*, *ADAMTS2*, *ZNF469* and *PRDM5*.		
EDS Kyphoscoliotic Type 1 (EDSKSCL1)	EDSKSCL1 (#225400) previously designated EDS6, is caused by homozygous or compound heterozygous mutation in the *PLOD1* (*153454) gene on chromosome 1p36. Inheritance is autosomal recessive.	*PLOD1* encodes a lysyl hydroxylase that catalyses the hydroxylation of lysine residues in X-lys-gly sequences of collagens and other proteins with collagen-like domains. This hydroxylation is essential for the stability of intermolecular collagen crosslinks.	EDSKSCL1 is characterized by skin fragility (easy bruising, friable skin, poor wound healing, widened atrophic scarring), scleral and ocular fragility/rupture, microcornea, facial dysmorphology. General features also include congenital muscle hypotonia, congenital or early onset kyphoscoliosis, joint hypermobility with subluxations or dislocations of shoulders, hips and knees [164].
EDS Kyphoscoliotic Type, 2 (EDSKSCL2)	EDSKSCL2 (#614557) is caused by homozygous or compound heterozygous mutations in *FKBP14* gene (*614505) on chromosome 7p15. Inheritance is autosomal recessive.	*FKBP14* is an ER-resident protein belonging to the family of FK506-binding peptidyl-prolyl *cis*–*trans* isomerases (PPIases). It catalyses the folding of COL3 and interacts with COL3, COL4 and COLX [165].	EDSKSCL2 is characterised by congenital hearing impairment (sensorineural, conductive, or mixed), follicular hyperkeratosis, muscle atrophy, bladder diverticula.
EDS dermatosparaxis Type (EDSDERMS)	EDSDERMS (#225410) is caused by mutation in *ADAMTS2* (*604539) gene on chromosome 5q35. Inheritance is autosomal recessive.	*ADAMTS2* encodes a procollagen protease that takes part to the processing of type I procollagen.	Dermatosparaxis means ‘tearing of skin.’ Patients present extreme skin laxity and fragility, easy bruising, extensive scar formation and joint laxity. Blue sclerae, micrognathia, umbilical hernia and postnatal growth retardation are reported [164].
Brittle Cornea Syndrome1 (BCS1)	BCS1 (#229200) can be caused by homozygous mutation in the *ZNF469* gene (*612078) on chromosome 16q24. Inheritance is autosomal recessive.	*ZNF469* encodes a zinc-finger protein that likely acts as a transcription factor or extra-nuclear regulator factor for the synthesis or organization of collagen fibres.	BCS1 and BCS2 are associated with retinal microvascular abnormalities, keratoconus or keratoglobus, blue sclerae, extreme corneal thinning and a high risk of corneal rupture. Hyperelasticity of the skin without excessive fragility and hypermobility of the joints are other hallmarks of the disease [164].
Brittle Cornea Syndrome2 (BCS2)	BCS2 (#614170) is caused by mutation in *PRDM5* gene (*614161) on chromosome 4q27. Inheritance is autosomal recessive.	PRDM5 seems to regulate the expression of proteins involved in extracellular matrix development and maintenance, including COL4A1 and COL11A1.	BCS2 features overlap with BCS1. Systemic abnormalities included increased skin laxity, pectus excavatum, scoliosis, congenital hip dislocation, recurrent shoulder dislocation, high-frequency hearing loss, high-arched palate and mitral valve prolapse [166].
CUTIS LAXA	Cutis laxa can be caused by mutations in either *PYCR1* (*179035) or *ALDH18A1* (#614438) gene.		Cutis laxa is a rare skin disorder characterized by wrinkled, redundant, inelastic and sagging skin due to defective synthesis of elastic fibres and other proteins of the ECM [167].
Cutis Laxa, autosomal recessive Type IIB (ARCL2B)	ARCL2B (#612940) is caused by homozygous or compound heterozygous mutation in the *PYCR1* gene (*179035) on chromosome 17q25.3. Inheritance is autosomal recessive.	*PYCR1* encodes the enzyme pyrroline-5-carboxylate reductase1, which catalyses the last step of proline synthesis. PYCR1 deficiency can affect the proper collagen formation.	ARCL2 is a more benign form of cutis laxa present at birth. Growth and developmental delay and skeletal anomalies are reported. Intellectual deficit and seizures have been reported in older patients [167]. Systemic manifestations are mild whereas pulmonary emphysema and cardiac anomalies are rare.
Cutis Laxa, autosomal recessive Type IIIB (ARCL3B)	ARCL3B (#614438) is caused by mutation in *PYCR1* gene (179035) on chromosome 17q25. Inheritance is autosomal recessive.		ARCL3B is a rare autosomal recessive disorder characterized by a progeria-like appearance with distinctive facial features, sparse hair, ophthalmologic abnormalities and intrauterine growth retardation [168].
Cutis Laxa, autosomal recessive, Type IIIA (ARCL3A)	ARCL3A (#219150) is caused by mutation in the *ALDH18A1* gene (*138250) on chromosome 10q24. Inheritance is autosomal recessive.	The protein encoded by *ALDH18A1* catalyses the reduction of glutamate to delta1-pyrroline-5-carboxylate, a critical step in the de novo biosynthesis of proline, ornithine and arginine.	ARCL3A is characterized by cutis laxa (a progeria-like appearance) and ophthalmologic abnormalities [169]. In some case, additional features have been described, including delayed development, intellectual disability, seizures and problems with movement that can worsen over time.
Cutis Laxa, autosomal dominant, Type III (ADCL3)	ADCL3 (#616603) is caused by mutation in *ALDH18A1* gene (*138250) on chromosome 10q24. Inheritance is autosomal dominant.		ADCL3 has a progeroid appearance characterized by thin skin with visible veins and wrinkles, ophthalmological abnormalities, clenched fingers, pre- and postnatal growth retardation and moderate intellectual disability. Patients also exhibit a combination of muscular hypotonia with brisk muscle reflexes [170].
Keratoconus-1 (KTCN1)	KTCN1 (#148300) is caused by heterozygous mutation in the *Visual system homeobox gene 1* (*VSX1,* *605020) gene on 20p11 chromosome. Inheritance is autosomal dominant.	*VSX1* encodes a homeoprotein that regulates the expression of the cone opsin genes early in development. Recent studies showed that the structural deformity of the cornea in KCTN patients may be due to reduced expression of collagens (*COL1A1* and *COL4A1*) and LOX family oxidases, as well as on the concomitant increased expression of *MMP9* [171].	KTCN1 is the most common corneal dystrophy. It is a bilateral, often asymmetrical, non-inflammatory progressive corneal ectasia that causes visual morbidity. In affected individuals, the cornea becomes progressively thin and conical in shape, resulting in myopia, irregular astigmatism and corneal scarring. It typically appears in the teenage years and then it progresses until the third and fourth decades. No specific treatment exists except corneal transplantation when visual acuity can no longer be corrected by contact lenses [172].

^a^ Information mainly based on OMIM, the Online Mendelian Index in Man at http://www.ncbi.nlm.nih.gov/entrez/query.fcgi?db=OMIM. Grey and white rows are used to distinguish the different disorders. Causative genes are in bold. Abbreviations: the acronyms of the pathologies are all specified inside the Table.
